# Quantification of renal function following stereotactic body radiotherapy for pancreatic cancer: secondary dosimetric analysis of a prospective clinical trial

**DOI:** 10.1186/s13014-017-0798-8

**Published:** 2017-04-27

**Authors:** Vivek Verma, Abhijeet R. Bhirud, Kyle A. Denniston, Nathan R. Bennion, Chi Lin

**Affiliations:** Department of Radiation Oncology, University of Nebraska Medical Center, 987521 Nebraska Medical Center, Ground Floor, Clarkson Tower, Omaha, NE 68198 USA

**Keywords:** Pancreatic cancer, Stereotactic body radiotherapy, Toxicity, Kidney, Glomerular filtration rate

## Abstract

**Background:**

This is the first known study examining renal function following stereotactic body radiotherapy (SBRT) for pancreatic head adenocarcinoma.

**Methods:**

Thirty-eight borderline-resectable/unresectable patients, part of an ongoing prospective trial, underwent 3 cycles of gemcitabine/5-fluorouracil followed by SBRT (5 daily fractions of 5/6/7/8 Gy) and concurrent nelfinavir. Thereafter, in resectable cases, surgery was performed within 4–8 weeks. The last available pre-SBRT creatinine was recorded, along with the highest post-SBRT value. Glomerular filtration rate (GFR) was calculated by the commonly-utilized Modification of Diet in Renal Disease formula. GFR decline was defined as the post-SBRT nadir GFR minus the pre-SBRT GFR. Correlations with the V5–V30, and mean/maximum kidney doses was performed. Statistics included Pearson correlation, Mann-Whitney, and Fisher’s exact tests.

**Results:**

The median total kidney volume was 355 cm^3^. Median dosimetric values were as follows: V5 (209 cm^3^), V10 (103 cm^3^), V15 (9 cm^3^), V20 (0 cm^3^), V25 (0 cm^3^); and mean (6.7 Gy) & maximum kidney dose (18.3 Gy). Median GFR change was −23 (range, −105 to 25) mL/min/1.73 cm^2^. Of all dosimetric parameters, only V5 was significantly associated with changes in GFR (Pearson *r* = −0.40, *p* = 0.012). In patients with V5 < 210 cm^3^, median GFR change was −11.8 mL/min/1.73 cm^2^, as compared with −37.1 mL/min/1.73 cm^2^ change in those with V5 ≥ 210 cm^3^ (*p* = 0.02). A GFR change < −23 mL/min/1.73 cm^2^ was observed in 6/20 (30%) patients with V5 < 210 cm^3^, versus 15/18 (83%) of those with V5 ≥ 210 cm^3^. Patients with V5 ≥ 210 cm^3^ were over ten times as likely to have GFR change < −23 mL/min/1.73 cm^2^ (*p* = 0.003). Using linear regression, GFR change ≈ −0.1748 × V5(cm^3^) + 8.63.

**Conclusions:**

In the first known analysis of renal function after pancreatic SBRT, evaluating patients on a prospective study, V5 ≥ 210 cm^3^ was associated with a post-SBRT GFR decline of >23 mL/min/1.73 cm^2^. If V5 is kept <210 cm^3^, median GFR decline was only 11.8 mL/min/1.73 cm^2^. Further validation is needed to ascertain definite dose-volume parameters and examine late renal decline.

## Background

The recent popularity of neoadjuvant stereotactic body radiotherapy (SBRT) for pancreatic cancer has created a necessity to better define dose-volume parameters in order to ensure the safety of nearby organs-at-risk (OARs) [1]. Although dose-volume constraints have been published for conventionally-fractionated radiotherapy, the relevance of these constraints is uncertain in the setting of SBRT [2, 3]. Although previously addressed for SBRT to renal tumors [4], kidney dosimetry in the pancreas SBRT setting has previously not been addressed, and dose-volume constraints of prior work (using various regimens) have not been correlated with renal function [5–9]. Though the irradiated renal volume is overall low in pancreatic SBRT (and recognizing that these data apply to pancreatic SBRT only), assessing the degree of post-SBRT renal impairment is important to provide a benchmark, especially as the use of pancreatic SBRT rises in the future. This is the first study to date examining this notion, evaluating patients enrolled on an ongoing prospective trial. In addition to radiation oncologists, these data are broadly applicable to medical oncologists, nephrologists, and/or general practitioners, all of whom may be involved in post-SBRT care of this population.

## Methods

This study was a retrospective secondary analysis of an ongoing prospective study of chemotherapy followed by SBRT and concurrent nelfinavir, a human immunodeficiency virus protease inhibitor harboring tumoricidal and radiosensitizing effects, observed in both preclinical studies [10], phase I trials [11], and phase II data [12]. All patients had biopsy-proven borderline resectable or unresectable pancreatic head adenocarcinoma without evidence of distant disease. Complete trial inclusion criteria are described elsewhere [13]; of note, maximum tumor dimension was <8 cm, serum creatinine ≤2 mg/dL, without prior abdominal radiotherapy.

Blood chemistries were obtained weekly during chemotherapy, and immediately prior to SBRT. Treatment course is also described elsewhere [13, 14], but consisted of 3 cycles of intravenous gemcitabine/5-fluorouracil/leucovorin. Subsequently, PET-CT scan, fiducial marker implantation, and MRI imaging was obtained. Four-dimensional CT (4D-CT) simulation (with obtainment of a free-breathing phase) was then performed, utilizing body fixation devices and intravenous/oral contrast, with no oral intake for several hours prior to simulation. The gross tumor volume was contoured with the assistance of PET-CT/MRI image fusion, and an internal target volume (ITV) was created to account for respiratory motion utilizing 4D-CT information. An isotropic 5 mm expansion was added to the ITV, forming the planning target volume (PTV). Uninvolved regional lymph nodes were not electively included in treatment volumes [6]. OARs were contoured in accordance with Radiation Therapy Oncology Group (RTOG) guidelines [15, 16]; the right and left kidneys were contoured individually and combined as a composite total kidney volume for the purposes of this study [8]. Though other dose constraints were from previous studies [5–9, 17] and RTOG 0631 [18], one-third of the total kidneys’ volumes were limited to <15 Gy [8]. As this was also a dose-escalation study, prescribed doses included 5, 6, 7, and 8 Gy in five daily fractions. The prescribed dose was required to cover 95% of the PTV at minimum. Accurate SBRT delivery was confirmed with daily image guidance using ExacTrac orthogonal kV images (Brainlab AG, Feldkirchen, Germany). Concurrent nelfinavir was administered with SBRT, which has hepatic metabolism and is not known to affect kidney function [19]. Thereafter, if deemed resectable on post-SBRT imaging, pancreaticoduodenectomy was performed. No patients received adjuvant systemic therapy.

Retrospectively, the last available pre-SBRT serum creatinine value (mg/dL) was recorded, along with post-SBRT values for the duration of patients’ follow-up/survival. For each creatinine value, the Modification of Diet in Renal Disease (MDRD) formula was used to calculate the corresponding glomerular filtration rate (GFR): 175 × Cr^−1.154^ × age^−0.203^ × 0.742(if female) × 1.210(if African-American). GFR change was calculated as the post-SBRT nadir GFR minus pre-SBRT GFR; negative values indicated a decline, positive values a rise. Though creatinine can indeed fluctuate in any patient, we deliberately analyzed based on the nadir GFR for this study, for multiple reasons. First, follow-up in the prospective trial was well-controlled in terms of post-SBRT/surgical medication changes, adjuvant therapies, and other items potentially influencing kidney function. Second, the methods were based on those in nephrologic literature, wherein the concept of peak creatinine/nadir GFR continues to gain popularity in follow-up for conditions having poor prognoses and/or frequent creatinine fluctuations [20, 21].

Dosimetric parameters analyzed for this study included V5 (cm^3^ of kidney receiving ≥5Gy)–V30, as well as mean and maximum kidney dose. Statistics were performed using SAS v.9.3 (Cary, NC). These included the Mann-Whitney & Fisher’s exact tests as non-parametric tests of comparing mean/maximum values and proportions, respectively, of several parameters analyzed herein. In order to associate GFR decline with dose-volume parameters, Pearson correlation was utilized; linear regression provided a numerical comparison between both variables.

## Results

Of 40 consecutively/prospectively-enrolled patients, two were excluded for not having available pre/post-SBRT creatinine values. Characteristics of the 38-patient study population is shown in Table 1. No patient underwent a change in dosage or new addition of anti-hypertensive medications known to affect the kidney. After SBRT, with mean 10-month (range, 0–26) follow-up, 30/38 (79%) patients experienced a rise in creatinine. Relatively consistently in all patients, GFR reached a nadir in an average of 5 months; median GFR change was −23 (range, −105–25) mL/min/1.73 m^2^. The median pre-SBRT GFR was 97 mL/min/1.73 m^2^, with a median nadir of 62 mL/min/1.73 m^2^ after SBRT (*p* < 0.001).

**Table 1:** Clinical characteristics of the study population. ECOG, Eastern Cooperative Oncology Group; SBRT, stereotactic body radiotherapy; GFR, glomerular filtration rate; PTV, planning target volume

Parameter	Value (Percent/Range)
Median (range) age, years	63 (35–80)
Gender	
Male	23 (60.5%)
Female	15 (39.5%)
ECOG performance status	
0	13 (34.2%)
1	20 (52.6%)
2	5 (13.2%)
3	0 (0%)
History of chronic kidney disease	
Yes	0 (0%)
No	38 (100%)
Nephroaffective medication at diagnosis	
Metformin	6 (16%)
ACE inhibitors	3 (8%)
Median (range) tumor size, cm	3.0 (0.8–6.5)
Resectability status	
Borderline resectable	13 (34.2%)
Unresectable	25 (65.8%)
Median (range) pre-SBRT creatinine, mg/dL	0.8 (0.5–1.4)
Median (range) pre-SBRT GFR, mL/min/1.73 m^2^	96.8 (50.1–142.0)
Median (range) post-SBRT creatinine peak, mg/dL	1.1 (0.5–3.57)
Median (range) pre-SBRT GFR nadir, mL/min/1.73 m^2^	61.6 (12.9–147.7)
Median (range) contoured volume of kidneys, cm^3^	355 (187–726)
Median (range) PTV volume, cm^3^	130 (59–275)
SBRT technique	
Fixed-beam	36 (94.7%)
Arc	2 (5.3%)
Receipt of pancreaticoduodenectomy	
Yes	10 (26.3%)
No	28 (73.7%)

Of the 26 patients who experienced a nadir GFR within 3 months, four patients experienced Common Toxicity Criteria for Adverse Events (CTCAE) grade 1 toxicity (creatinine 1.5–2 times above baseline), grade 2 (2–3 times above baseline) in one patient, and grade 3 (creatinine over three times baseline and/or over 4 mg/dL) in two patients (neither required hospitalization). In the 12 patients whose GFR nadir occurred more chronically (after 3 months), six patients were categorized as grade 1 renal toxicity, five patients grade 2, and one patient grade 3. No patient required dialysis. At last follow-up however, five patients persisted with grade 1 renal toxicity and the remainder had no CTCAE-defined toxicities. Regarding post-nadir trends, by last follow-up, creatinine was stable (within 20% of baseline [22–24]) in 17 patients, increased in 15 patients, and decreased in six patients.

**Fig. 1:**
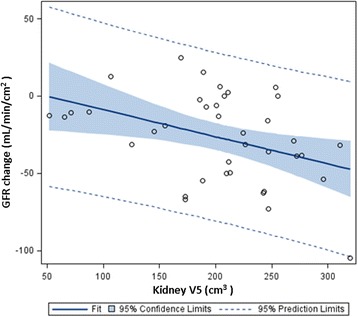
Scatter plot demonstrating that change in glomerular filtration rate (GFR) after pancreas stereotactic radiotherapy is inversely related to kidney V5

Median (range) values for V5, V10, V15, V20, and V25 were 209 (52–320), 103 (0–242), 9 (0–110), 0 (0–22), and 0 (0–5) cm^3^ respectively. Median values of kidney mean and maximum doses were 6.7 and 18.3 Gy, respectively. Though V10–V30, age, gender, baseline GFR, tumor resection, and postoperative chemotherapy did not correlate with GFR decline (all *p* > 0.05), V5 correlated significantly (Pearson *r* = −0.40, *p* = 0.012; Fig. 1). Hence, using linear regression, GFR change roughly equated to: (−0.1748 × V5) + 8.63.

A GFR change < −23 mL/min/1.73 cm^2^ was observed in 6/20 (30%) patients with V5 < 210 cm^3^, versus 15/18 (83%) of those with V5 ≥ 210 cm^3^. Moreover, in patients with V5 < 210 cm^3^, median GFR change was −11.8 mL/min/1.73 cm^2^, as compared with −37.1 mL/min/1.73 cm^2^ change in those with V5 ≥ 210 cm^3^ (*p* = 0.02). Patients with V5 ≥ 210 cm^3^ were vastly more likely to have GFR change < −23 mL/min/1.73 cm^2^ (*p* = 0.003).

## Discussion

In summary, these data relate kidney V5 to renal GFR decline after pancreas SBRT. By retrospectively analyzing data obtained as part of a prospective trial, we provide the first known association between kidney doses in pancreatic SBRT and renal dysfunction. It should be mentioned that these data are applicable to pancreatic SBRT and not necessarily to SBRT for other intra-abdominal areas. Nevertheless, our results underscore the need to control low-dose spillage to the renal system.

There are salient reflections from these data. It is most likely that V5, but not other higher-dose parameters, correlated most with the renal endpoints herein, because of the higher amount of low-dose spillage to the kidneys with SBRT, which notably depends also on treatment technique. This is consistent with comparatively fewer differences between patients in V10 and higher values, whereas V5 was most liable to be different between patients. This is a prime reason our data may not apply to primary renal neoplasms, which are associated with higher renal doses from closer anatomic proximity, as opposed to the largely lower kidney doses delivered during pancreas SBRT. However, it does not rule out the fact that both high-dose as well as low-dose areas could impact kidney function. Nevertheless, a message of our results is that pancreas SBRT is not likely to deliver high renal doses, thus decreasing the likelihood that high-dose areas would find to correlate with renal decline.

Our data will next be compared to existing literature. Currently, only one other study has published data on observed kidney doses, but without correlation to renal function; this study also used an uncommon, and therefore potentially less generalizable, single-fraction SBRT technique [25]. This study complements a recently published report of renal decline after SBRT of renal cell carcinoma, although clinical and radiotherapeutic circumstances of both studies were substantially different [4]. That study utilized a substantial proportion of single-fraction treatment to the kidney, as opposed to 5-fraction treatment relatively away from the kidney. Hence, the finding therein of higher (>50% isodose line) renal doses correlating with renal decline would naturally not be congruent with this study (which demonstrated similar findings with lower-dose volumes). Nevertheless, regarding pancreatic SBRT, Table 2 displays various other kidney constraints utilized in five available investigations. With further research, these constraints may be better standardized in the future.

**Table 2:** Selected existing reports describing dose constraints to the kidney during pancreatic SBRT. SBRT, stereotactic body radiotherapy; SIB, simultaneous integrated boost

Study	Treatment regimen/dose	Dose-volume constraint
Koong et al. [4]	45 Gy/25 fractions + 25 Gy single-fraction SBRT	70% of each kidney <15 Gy
Chang et al. [5]	79% patients with 25 Gy single-fraction SBRT; 21% patients with preceding 45–54 Gy/25–30 fractions	75% of each kidney <5 Gy
Chuong et al. [6]	25–30 Gy/5 fractions, SIB to involved perivascular areas to 35–50 Gy	Kidney mean dose <10 Gy
Su et al. [7]	30–36 Gy/3 fractions	1/3 of kidney volume <15 Gy
Herman et al. [8]	33 Gy/5 fractions	75% of both kidneys <12 Gy

Regarding strengths and weaknesses of these data, this investigation is part of a prospective trial and the associated standardization of treatment, thus avoiding potential bias associated with retrospective data analysis. It should be mentioned, however, that this endpoint was planned to be analyzed but not specifically listed as a pre-specified endpoint of the trial. Moreover, confirmation of our methods from the nephrology perspective is noteworthy. Chiefly, though creatinine can indeed fluctuate in various individuals, as mentioned before, there were multiple reasons for deliberately measuring peak creatinine/nadir GFR (as opposed to temporal patterns) including being an increasingly-utilized option in patients with poor prognosis [20, 21]. Additionally, although there are several methods to calculate GFR, each with corresponding strengths and weaknesses, the MDRD is used for patients both with normal GFR and renal disease, and has been validated in large cohorts [26]. Though the MDRD formula does not adjust for body mass, any formula has similar shortcomings. Lastly, though a GFR drop of 23 mL/min/1.73 cm^2^ (the median value) was utilized as a comparative “standard”, high-quality data have demonstrated its value. In patients with similar baseline creatinine, a drop in 35 mL/min/1.73 cm^2^ is associated with increased all-cause and cardiovascular mortality [27]. Hence, in practice, GFR drops between 20 and 50 mL/min/1.73 cm^2^ are considered “clinically noteworthy” endpoints. Additionally, it has been recommended to grade kidney injury using GFR changes, and a change of 23 mL/min/1.73 cm^2^ roughly corresponds with a one-level upstaging of chronic kidney disease (CKD) [3]. For these several reasons, we strongly posit that our median value cutoff is quite clinically applicable. In addition to for non-pancreas SBRT cases, our data have limited applicability to patients with acute kidney injury (AKI) or CKD; though no patients had CKD, presence of occult AKI can never be ruled out. Additionally, it is difficult and impractical to control the numerous factors that can influence creatinine readings at any given time. Additionally, though comparison was made to one pre-SBRT “initial” GFR datapoint, utilizing creatinine measurements during chemotherapy can be misleading owing to frequent receipt of intravenous hydration (or drug infusion itself). Moreover, though these data have the advantage of detailing the temporal relationship of post-SBRT GFR decline, it may not substitute for individual creatinine/GFR measurements immediately prior to interventions impacting the kidneys. The vast majority of patients did not have individual renal scans in order to verify correlation with both dosimetry and calculated GFR. Next, that some patients had surgery may impact post-SBRT creatinine values; however, this was in a minority of patients (*n* = 10), none of which displayed outliers in renal measurements for the study period. Furthermore, it was not feasible to control for every potential medication that may have effects on the kidney. Additionally, our contouring of both kidneys as one structure, instead of proportional/spared volume or other parameters, is consistent with other sources [7–9]. However, it is acknowledged that kidney motion was not controlled using abdominal compression, because dose to the renal apices may be different than to the central parenchyma. Furthermore, the planar imaging alignment assumes that the planned dose delivered to the kidneys equates to the actual delivered dose, which like other studies may not be accurate because kidney location in the field and/or with respect to fiducials cannot be precisely verified in every fraction. Taken together, strengths and weaknesses are described when placing these results in context of existing and future work.

In summary, the value of kidney V5 in clinically-evident renal function decline must be validated in an independent cohort (and in non-SBRT cases), as these data are hypothesis-generating. Additionally, though this study measured a GFR nadir and did not seek to examine a prolonged time course of post-SBRT GFR decline, this can indeed occur after SBRT [28]; unfortunately, measurement of longer-term data is difficult owing to the abysmal survival of this disease, and our short follow-up was severely limited by patient survival [29]. Data examining the time course of GFR decline for SBRT-induced kidney injury, even in the short-term, are warranted.

## Conclusions

In the first known analysis of renal function after pancreatic SBRT, evaluating patients on a prospective study, total kidney V5 ≥ 210 cm^3^ was associated with a post-SBRT GFR decline of >23 mL/min/1.73 cm^2^. If V5 is kept <210 cm^3^, median GFR decline was only 11.8 mL/min/1.73 cm^2^. Further verification of these data is needed to better ascertain dose-volume relationships with GFR, and examine late renal decline.

## References

[1] Verma V, Li J, Lin C. Neoadjuvant Therapy for Pancreatic Cancer: Systematic Review of Postoperative Morbidity, Mortality, and Complications. Am J Clin Oncol. 2016;39:302–13. 10.1097/COC.0000000000000278 26950464

[2] Marks LB, Yorke ED, Jackson A (2010). The use of normal tissue complication probability (NTCP) models in the clinic. Int J Radiat Oncol Biol Phys.

[3] Dawson LA, Kavanagh BD, Paulino AC (2010). Radiation-associated kidney injury. Int J Radiat Oncol Biol Phys.

[4] Siva S, Jackson P, Kron T (2016). Impact of stereotactic radiotherapy on kidney function in primary renal cell carcinoma: establishing a dose-response relationship. Radiother Oncol.

[5] Koong AC, Christofferson E, Le QT (2005). Phase II study to assess the efficacy of conventionally fractionated radiotherapy followed by a stereotactic radiosurgery boost in patients with locally advanced pancreatic cancer. Int J Radiat Oncol Biol Phys.

[6] Chang DT, Schellenberg D, Shen J (2009). Stereotactic radiotherapy for unresectable adenocarcinoma of the pancreas. Cancer.

[7] Chuong MD, Springett GM, Freilich JM (2013). Stereotactic body radiation therapy for locally advanced and borderline resectable pancreatic cancer is effective and well-tolerated. Int J Radiat Oncol Biol Phys.

[8] Su TS, Liang P, Lu HZ (2015). Stereotactic body radiotherapy using CyberKnife for locally advanced unresectable and metastatic pancreatic cancer. World J Gastroenterol.

[9] Herman JM, Chang DT, Goodman KA (2015). Phase 2 Multi-institutional Trial Evaluating Gemcitabine and Stereotactic Body Radiotherapy for Patients With Locally Advanced Unresectable Pancreatic Adenocarcinoma. Cancer.

[10] Gupta AK, Cerniglia GJ, Mick R (2005). HIV protease inhibitors block Akt signaling and radiosensitize tumor cells both in vitro and in vivo. Cancer Res.

[11] Brunner TB, Geiger M, Grabenbauer GG (2008). Phase I trial of the human immunodeficiency virus protease inhibitor nelfinavir and chemoradiation for locally advanced pancreatic cancer. J Clin Oncol.

[12] Wilson JM, Fokas E, Dutton SJ (2016). ARCII: a phase II trial of the HIV protease inhibitor Nelfinavir in combination with chemoradiation for locally advanced inoperable pancreatic cancer. Radiother Oncol.

[13] NCT01068327. Stereotactic Radiation Therapy, Nelfinavir Mesylate, Gemcitabine Hydrochloride, Leucovorin Calcium, and Fluorouracil in Treating Patients With Locally Advanced Pancreatic Cancer. https://clinicaltrials.gov/ct2/show/NCT01068327. Accessed 23 May 2016.

[14] Verma V, Lazenby AJ, Zheng D, et al. Dosimetric parameters correlate with duodenal histopathologic damage after stereotactic body radiotherapy for pancreatic cancer: secondary analysis of a prospective clinical trial. Radiother Oncol. 2017;122:464-69. 10.1016/j.radonc.2016.12.030 PMC5346338 28089484

[15] Radiation Therapy Oncology Group. Upper abdominal normal organ contouring consensus guidelines. https://www.rtog.org/CoreLab/ContouringAtlases/UpperAbdominalNormalOrganContouringConsensusGuidelines.aspx. Accessed 23 May 2016. 10.1016/j.prro.2013.06.004 PMC4285338 24890348

[16] Jabbour SK, Hashem SA, Bosch W (2014). Upper abdominal normal organ contouring guidelines and atlas: a Radiation Therapy Oncology Group consensus. Pract Radiat Oncol.

[17] Grimm J, LaCouture T, Croce R (2011). Dose tolerance limits and dose volume histogram evaluation for stereotactic body radiotherapy. J Appl Clin Med Phys.

[18] Radiation Therapy Oncology Group. RTOG 0631 Protocol Information. https://www.rtog.org/ClinicalTrials/ProtocolTable/StudyDetails.aspx?study=0631. Accessed 23 May 2016.

[19] Boubaker K, Sudre P, Bally F (1998). Changes in renal function associated with indinavir. AIDS.

[20] Angeli P, Gines P, Wong F (2015). Diagnosis and management of acute kidney injury in patients with cirrhosis: revised consensus recommendations of the International Club of Ascites. J Hepatol.

[21] Broce JC, Price LL, Liangos O (2011). Hospital-acquired acute kidney injury: an analysis of nadir-to-peak serum creatinine increments stratified by baseline estimated GFR. Clin J Am Soc Nephrol.

[22] Paige NM, Nagami GT (2009). The top 10 things nephrologists wish every primary care physician knew. Mayo Clin Proc.

[23] The Renal Association. About eGFR. http://www.renal.org/information-resources/the-uk-eckd-guide/about-egfr#sthash.RpsAXiu1.dpbs. Accessed 17 Feb 2017.

[24] Weber CL, Beaulieu M, Karr G, Levin A (2008). Demystifying chronic kidney disease: clinical caveats for the family physician. BC Med J.

[25] Koong AC, Le QT, Ho A (2004). Phase I study of stereotactic radiosurgery in patients with locally advanced pancreatic cancer. Int J Radiat Oncol Biol Phys.

[26] Levey AS, Bosch JP, Lewis JB (1999). A more accurate method to estimate glomerular filtration rate from serum creatinine: a new prediction equation. Ann Intern Med.

[27] Chronic Kidney Disease Prognosis Consortium (2010). Association of estimated glomerular filtration rate and albuminuria with all-cause and cardiovascular mortality in general population cohorts: a collaborative meta-analysis. Lancet.

[28] Teh B, Bloch C, Galli-Guevara M (2007). The treatment of primary and metastatic renal cell carcinoma (RCC) with image-guided stereotactic body radiation therapy (SBRT). Biomed Imaging Intervion J.

[29] Elhammali A, Patel M, Weinberg B (2015). Late gastrointestinal tissue effects after hypofractionated radiation therapy of the pancreas. Radiat Oncol.

